# Diaqua­(2,2′-bipyridine-κ^2^ *N*,*N*′)bis­(perchlorato-κ*O*)copper(II)

**DOI:** 10.1107/S1600536811013808

**Published:** 2011-04-16

**Authors:** Maamar Damous, Meriem Hamlaoui, Sofiane Bouacida, Hocine Merazig, Jean-Claude Daran

**Affiliations:** a Unité de Recherche de Chimie de l’Environnement et Moléculaire Structurale, CHEMS, Université Mentouri-Constantine, 25000 Algeria; b Laboratoire de Chimie de Coordination, UPR CNRS 8241, 205 Route de Narbonne, 31077 Toulouse Cedex, France

## Abstract

The central CuN_2_O_4_ motif of the title compound, [Cu(ClO_4_)_2_(C_10_H_8_N_2_)(H_2_O)_2_], exhibits a Jahn–Teller-distorted octa­hedral geometry around the metal atom, showing a considerably long Cu—O bond distance of 2.5058 (12) Å towards the second perchlorate group, giving a (4 + 1+1)-type coordination mode. In the crystal, the components are linked *via* inter­molecular O—H⋯O hydrogen bonds, forming layers parallel to (001). Additional stabilization within these layers is provided by π–π [centroid–centroid distances of 3.7848 (9)–4.4231 (9) Å] stacking inter­actions.

## Related literature

For applications of related compounds, see: Kurzak *et al.* (1999 ▶). For the coordination spheres of copper in related compounds, see: Hathaway (1973 ▶). For hydrogen-bond motifs, see: Bernstein *et al.* (1995 ▶); Etter *et al.* (1990 ▶). 
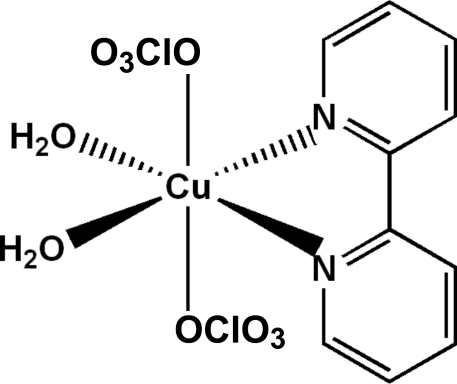


## Experimental

### 

#### Crystal data

[Cu(ClO_4_)_2_(C_10_H_8_N_2_)(H_2_O)_2_]
*M* *_r_* = 454.67
Monoclinic, 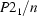

*a* = 7.1378 (4) Å
*b* = 12.7853 (7) Å
*c* = 16.8033 (11) Å
β = 92.025 (6)°
*V* = 1532.49 (16) Å^3^
*Z* = 4
Mo *K*α radiation
μ = 1.83 mm^−1^
*T* = 296 K
0.13 × 0.07 × 0.05 mm

#### Data collection

Oxford Diffraction Xcalibur Sapphire2 diffractometer
Absorption correction: multi-scan (*CrysAlis RED*; Oxford Diffraction, 2008 ▶) *T*_min_ = 0.580, *T*_max_ = 1.000
16487 measured reflections
5135 independent reflections
4239 reflections with *I* > 2σ(*I*)
*R*_int_ = 0.034

#### Refinement

*R*[*F* ^2^ > 2σ(*F* ^2^)] = 0.030
*wR*(*F* ^2^) = 0.080
*S* = 1.05
5135 reflections
238 parameters
H atoms treated by a mixture of independent and constrained refinement
Δρ_max_ = 0.42 e Å^−3^
Δρ_min_ = −0.63 e Å^−3^

### 

Data collection: *CrysAlis CCD* (Oxford Diffraction, 2008 ▶); cell refinement: *CrysAlis CCD*; data reduction: *CrysAlis CCD*; program(s) used to solve structure: *SIR2002* (Burla *et al.*, 2003 ▶); program(s) used to refine structure: *SHELXL97* (Sheldrick, 2008 ▶); molecular graphics: *ORTEP-3 for Windows* (Farrugia, 1997 ▶) and *DIAMOND* (Brandenburg & Berndt, 2001 ▶); software used to prepare material for publication: *WinGX* (Farrugia, 1999 ▶).

## Supplementary Material

Crystal structure: contains datablocks global, I. DOI: 10.1107/S1600536811013808/bq2292sup1.cif

Structure factors: contains datablocks I. DOI: 10.1107/S1600536811013808/bq2292Isup2.hkl

Additional supplementary materials:  crystallographic information; 3D view; checkCIF report

## Figures and Tables

**Table 1:** Hydrogen-bond geometry (Å, °)

*D*—H⋯*A*	*D*—H	H⋯*A*	*D*⋯*A*	*D*—H⋯*A*
O1*W*—H1*W*⋯O10^i^	0.72 (2)	2.04 (2)	2.7078 (17)	155 (3)
O1*W*—H2*W*⋯O3^ii^	0.88 (2)	1.89 (2)	2.7665 (17)	177.3 (18)
O2*W*—H3*W*⋯O3^iii^	0.76 (2)	2.13 (2)	2.8802 (18)	169 (2)
O2*W*—H4*W*⋯O4	0.78 (2)	2.37 (2)	2.9518 (19)	133 (2)
O2*W*—H4*W*⋯O7^iv^	0.78 (2)	2.26 (3)	2.8349 (18)	132 (2)

Symmetry codes: (i) 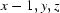; (ii) 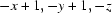; (iii) 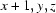; (iv) 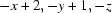.

**Fig. 1.**
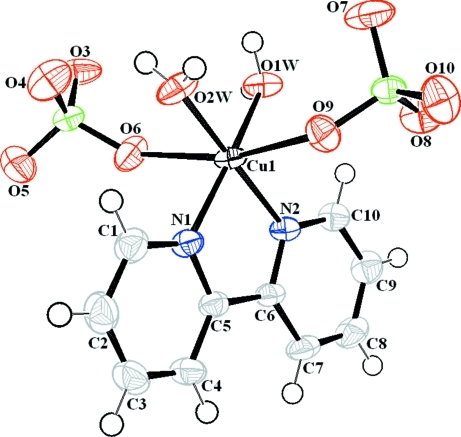
(Farrugia, 1997) The asymmetric unit of the title compound with the atomic labeling scheme. Displacements are drawn at the 50% probability level.

**Fig. 2.**
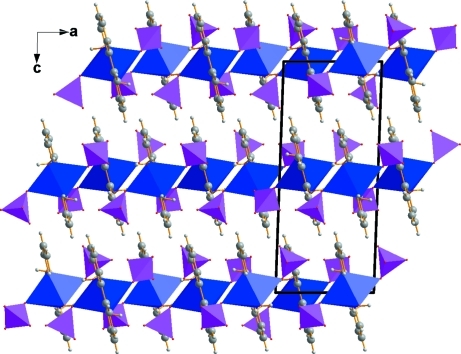
(Brandenburg & Berndt, 2001) A diagram of the layered crystal packing in (I), viewed down the b axis, showing layers parallel to (001) with alterning polyhedrals (ClO4 and CuN2O4).

**Fig. 3.**
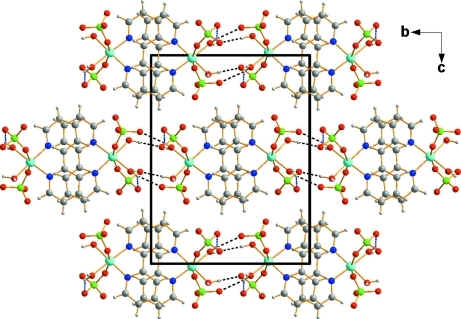
(Brandenburg & Berndt, 2001) A part of crystal packing of (I) showing hydrogen bond connections in the same layer as dashed line.

## supplementary crystallographic information

### Comment

Copper(II) complexes containing O, N-donor atoms are very important owing to their significant catalytic activity in the preparative oxygenation of phenols and other substances, and their significant antibacterial and anticancer activity (Kurzak *et al.*, 1999).

The asymmetric unit of (I), and the atomic numbering used, is illustrated in Fig. 1. The Cu^II^ atom is located in a Jahn-Teller distorted octahedral coordination environment with two N atoms from one 2,2'-bipyridine ligand (N1, N2) (*d*(Cu—N) = 1.9723 (13)–1.9805 (12) Å) and two O atoms from two water molecular adopting a planar arrangement (*d*(Cu—O) = 1.9621 (12)–1.9719 (12) Å). The Cu(II) center is displaced out of the N_2_O_2_ plane by 0.028 (2)Å in the direction of one of perchlorate ligand with d(Cu—O9) = 2.3287 (12) Å. The O atom of the second perchlorate group occupies a sixth coordination site at a longer distance of 2.5058 (12) Å, completing the overall (4 + 1 + 1) type coordination. O9 is situated slightly off the axial direct of the square pyramid, nevertheless it is close enough to the Cu atom (Hathaway, 1973). The bipyridine rings of the 2,2'-bipyridine ligand are twisted relative to each other at 2.2 (8)°.

The crystal structure can be described as alternating layers of polyhedral (ClO_4_ tetrahedrals and CuN_2_O_4_ octahedrals) perpendicular to *c* axis (Fig. 2).

The crystal packing in (I) is governed by classical hydrogen bond,*viz*. water molecules and perchlorate (Table 1, Fig. 3). All water H atoms are involved in these hydrogen bonds In the crystal, the components of the structure are linked *via* intermolecular O—H···O hydrogen bonds to form a two-dimensional layers parallel to (001) plane (Fig. 3). Additional stabilization within these layers is provided by π–π [3.7848 (9)Å to 4.4231 (9) Å] stacking interactions. These interaction bonds link the molecules within the layers and also link the layers together and reinforcing the cohesion of the structure.

The combination of these hydrogen bonds generates an alternating centrosymmetric rings in two-dimensional network which can be described by the graph-set motif *R*_4_^2^(12) and *R*_4_^4^(16) (Bernstein *et al.* 1995; Etter *et al.*, 1990).

### Experimental

The title compound was prepared by adding a methanol solution (10 ml) of copper (II) acetate monohydrate (0.1 mmol) to a methanol solution (10 ml) of 2,2'-bipyridine (0.1 mmol) and (1 ml) the perchloric acid. The mixture was stirred for about 2 h at 323 K and filtered. The filtrate was slowly evaporated at room temperature to yield blue crystals of (I) suitable for X-ray analysis.

### Refinement

H atoms of water molecule were located in difference Fourier maps and refined isotropically using restraints *U*_iso_(H) = 1.5*U*_eq_(O). The remaining H atoms were localized on Fourier maps but introduced in calculated positions and treated as riding on their parent atoms (C_aryl_) with C_aryl_—H_aryl_=0.93Å and *U*_iso_(H_aryl_)=1.2*U*_eq_(C_aryl_).

### Crystal data

**Table:**

[Cu(ClO_4_)_2_(C_10_H_8_N_2_)(H_2_O)_2_]	*F*(000) = 916
*M**_r_* = 454.67	*D*_x_ = 1.971 Mg m^−^^3^
Monoclinic, *P*2_1_/*n*	Mo *K*α radiation, λ = 0.7107 Å
*a* = 7.1378 (4) Å	Cell parameters from 9763 reflections
*b* = 12.7853 (7) Å	θ = 3.0–32.3°
*c* = 16.8033 (11) Å	µ = 1.83 mm^−^^1^
β = 92.025 (6)°	*T* = 296 K
*V* = 1532.49 (16) Å^3^	Needle, blue
*Z* = 4	0.13 × 0.07 × 0.05 mm

### Data collection

**Table:**

Oxford Diffraction Xcalibur Sapphire2 diffractometer	5135 independent reflections
Radiation source: Enhance (Mo) X-ray Source	4239 reflections with *I* > 2σ(*I*)
graphite	*R*_int_ = 0.034
Detector resolution: 8.2632 pixels mm^-1^	θ_max_ = 32.3°, θ_min_ = 3.1°
ω scans	*h* = −10→6
Absorption correction: multi-scan (*CrysAlis RED*; Oxford Diffraction, 2008)	*k* = −19→19
*T*_min_ = 0.580, *T*_max_ = 1.000	*l* = −25→25
16487 measured reflections	

### Refinement

**Table:**

Refinement on *F*^2^	Primary atom site location: structure-invariant direct methods
Least-squares matrix: full	Secondary atom site location: difference Fourier map
*R*[*F*^2^ > 2σ(*F*^2^)] = 0.030	Hydrogen site location: inferred from neighbouring sites
*wR*(*F*^2^) = 0.080	H atoms treated by a mixture of independent and constrained refinement
*S* = 1.05	*w* = 1/[σ^2^(*F*_o_^2^) + (0.0469*P*)^2^] where *P* = (*F*_o_^2^ + 2*F*_c_^2^)/3
5135 reflections	(Δ/σ)_max_ = 0.001
238 parameters	Δρ_max_ = 0.42 e Å^−^^3^
0 restraints	Δρ_min_ = −0.63 e Å^−^^3^

### Special details

**Table:**

Experimental. CrysAlis RED, Oxford Diffraction Ltd. (Empirical absorption correction using spherical harmonics, implemented in SCALE3 ABSPACK scaling algorithm).
Geometry. All e.s.d.'s (except the e.s.d. in the dihedral angle between two l.s. planes) are estimated using the full covariance matrix. The cell e.s.d.'s are taken into account individually in the estimation of e.s.d.'s in distances, angles and torsion angles; correlations between e.s.d.'s in cell parameters are only used when they are defined by crystal symmetry. An approximate (isotropic) treatment of cell e.s.d.'s is used for estimating e.s.d.'s involving l.s. planes.
Refinement. Refinement of *F*^2^ against ALL reflections. The weighted *R*-factor *wR* and goodness of fit *S* are based on *F*^2^, conventional *R*-factors *R* are based on *F*, with *F* set to zero for negative *F*^2^. The threshold expression of *F*^2^ > σ(*F*^2^) is used only for calculating *R*-factors(gt) *etc*. and is not relevant to the choice of reflections for refinement. *R*-factors based on *F*^2^ are statistically about twice as large as those based on *F*, and *R*- factors based on ALL data will be even larger.

### Fractional atomic coordinates and isotropic or equivalent isotropic displacement parameters (Å^2^)

**Table:**

	*x*	*y*	*z*	*U*_iso_*/*U*_eq_	
C1	0.8677 (2)	0.16080 (13)	0.14228 (10)	0.0255 (3)	
H1	0.8893	0.2282	0.1613	0.031*	
C2	0.8928 (3)	0.07755 (15)	0.19373 (11)	0.0302 (4)	
H2	0.9318	0.0886	0.2465	0.036*	
C3	0.8593 (3)	−0.02215 (14)	0.16589 (11)	0.0299 (4)	
H3	0.8759	−0.0794	0.1995	0.036*	
C4	0.8012 (2)	−0.03613 (13)	0.08784 (11)	0.0263 (3)	
H4	0.7765	−0.1029	0.0681	0.032*	
C5	0.7797 (2)	0.05028 (11)	0.03897 (10)	0.0189 (3)	
C6	0.7203 (2)	0.04419 (11)	−0.04548 (9)	0.0181 (3)	
C7	0.6861 (2)	−0.04880 (12)	−0.08553 (11)	0.0244 (3)	
H7	0.6975	−0.1125	−0.059	0.029*	
C8	0.6349 (2)	−0.04559 (13)	−0.16509 (11)	0.0283 (4)	
H8	0.6092	−0.1071	−0.1929	0.034*	
C9	0.6220 (2)	0.04947 (14)	−0.20331 (11)	0.0269 (3)	
H9	0.589	0.0529	−0.2573	0.032*	
C10	0.6588 (2)	0.13967 (12)	−0.16024 (10)	0.0226 (3)	
H10	0.6518	0.2039	−0.1861	0.027*	
N1	0.81339 (18)	0.14762 (10)	0.06591 (8)	0.0196 (2)	
N2	0.70407 (17)	0.13722 (9)	−0.08243 (8)	0.0186 (2)	
O1W	0.66836 (17)	0.36086 (9)	−0.09192 (8)	0.0237 (2)	
H1W	0.568 (3)	0.3587 (18)	−0.0987 (14)	0.036*	
H2W	0.691 (3)	0.4275 (19)	−0.0820 (14)	0.036*	
O2W	0.86002 (18)	0.37137 (9)	0.05755 (9)	0.0274 (3)	
H3W	0.958 (3)	0.394 (2)	0.0544 (15)	0.041*	
H4W	0.805 (3)	0.415 (2)	0.0787 (15)	0.041*	
O3	0.24885 (18)	0.43183 (9)	0.05923 (9)	0.0361 (3)	
O4	0.51492 (18)	0.41771 (11)	0.14266 (8)	0.0363 (3)	
O5	0.2632 (2)	0.30038 (12)	0.15559 (9)	0.0406 (3)	
O6	0.45262 (17)	0.28891 (10)	0.04596 (8)	0.0313 (3)	
O7	1.1044 (2)	0.43059 (10)	−0.13244 (9)	0.0389 (3)	
O8	1.02596 (19)	0.27740 (10)	−0.20091 (8)	0.0322 (3)	
O9	1.06594 (16)	0.27377 (10)	−0.06166 (7)	0.0256 (2)	
O10	1.32354 (16)	0.29465 (11)	−0.14184 (8)	0.0324 (3)	
Cu1	0.76523 (3)	0.258975 (14)	−0.013428 (12)	0.01792 (6)	
Cl1	0.37148 (5)	0.35902 (3)	0.10177 (2)	0.02055 (8)	
Cl2	1.12857 (5)	0.32027 (3)	−0.13482 (2)	0.01947 (8)	

### Atomic displacement parameters (Å^2^)

**Table:**

	*U*^11^	*U*^22^	*U*^33^	*U*^12^	*U*^13^	*U*^23^
C1	0.0304 (8)	0.0241 (8)	0.0220 (8)	0.0019 (6)	−0.0012 (7)	−0.0029 (6)
C2	0.0336 (9)	0.0365 (9)	0.0204 (8)	0.0073 (7)	0.0003 (7)	0.0032 (7)
C3	0.0333 (9)	0.0288 (8)	0.0279 (9)	0.0083 (7)	0.0037 (7)	0.0103 (7)
C4	0.0307 (8)	0.0175 (7)	0.0308 (9)	0.0039 (6)	0.0037 (7)	0.0051 (6)
C5	0.0184 (6)	0.0152 (6)	0.0233 (7)	0.0020 (5)	0.0016 (6)	0.0006 (5)
C6	0.0178 (6)	0.0126 (6)	0.0237 (7)	−0.0008 (5)	0.0010 (5)	−0.0004 (5)
C7	0.0276 (8)	0.0130 (6)	0.0326 (9)	−0.0012 (6)	0.0015 (7)	−0.0030 (6)
C8	0.0293 (8)	0.0220 (7)	0.0334 (9)	−0.0058 (6)	0.0005 (7)	−0.0109 (7)
C9	0.0281 (8)	0.0286 (8)	0.0239 (8)	−0.0041 (6)	−0.0028 (6)	−0.0053 (6)
C10	0.0230 (7)	0.0215 (7)	0.0230 (8)	−0.0012 (6)	−0.0027 (6)	0.0001 (6)
N1	0.0219 (6)	0.0160 (5)	0.0209 (6)	0.0015 (5)	−0.0011 (5)	−0.0007 (5)
N2	0.0195 (6)	0.0139 (5)	0.0222 (6)	−0.0011 (4)	−0.0013 (5)	0.0000 (4)
O1W	0.0211 (5)	0.0150 (5)	0.0345 (7)	−0.0007 (4)	−0.0064 (5)	0.0023 (4)
O2W	0.0233 (6)	0.0193 (6)	0.0394 (7)	0.0001 (4)	−0.0027 (5)	−0.0115 (5)
O3	0.0356 (7)	0.0183 (6)	0.0527 (9)	0.0008 (5)	−0.0205 (6)	0.0035 (5)
O4	0.0308 (6)	0.0373 (7)	0.0398 (8)	−0.0013 (6)	−0.0138 (6)	−0.0121 (6)
O5	0.0461 (8)	0.0378 (8)	0.0386 (8)	−0.0010 (6)	0.0145 (7)	0.0068 (6)
O6	0.0275 (6)	0.0290 (6)	0.0379 (7)	−0.0014 (5)	0.0065 (5)	−0.0122 (5)
O7	0.0552 (9)	0.0157 (6)	0.0452 (8)	0.0009 (6)	−0.0055 (7)	−0.0011 (5)
O8	0.0338 (7)	0.0316 (6)	0.0302 (7)	−0.0026 (5)	−0.0140 (6)	−0.0030 (5)
O9	0.0221 (5)	0.0294 (6)	0.0254 (6)	−0.0003 (5)	0.0015 (5)	0.0040 (5)
O10	0.0187 (5)	0.0460 (8)	0.0325 (7)	0.0012 (5)	0.0009 (5)	0.0046 (6)
Cu1	0.02028 (10)	0.01089 (9)	0.02227 (10)	−0.00087 (6)	−0.00360 (7)	−0.00050 (6)
Cl1	0.02118 (16)	0.01651 (15)	0.02369 (18)	0.00116 (12)	−0.00293 (13)	−0.00168 (13)
Cl2	0.01900 (16)	0.01621 (15)	0.02290 (17)	−0.00066 (12)	−0.00351 (13)	0.00004 (12)

### Geometric parameters (Å, °)

**Table:**

C1—N1	1.338 (2)	C10—N2	1.336 (2)
C1—C2	1.379 (2)	C10—H10	0.93
C1—H1	0.93	N1—Cu1	1.9723 (13)
C2—C3	1.376 (3)	N2—Cu1	1.9805 (12)
C2—H2	0.93	O1W—Cu1	1.9621 (12)
C3—C4	1.373 (3)	O2W—Cu1	1.9719 (12)
C3—H3	0.93	O1W—H1W	0.72 (2)
C4—C5	1.382 (2)	O1W—H2W	0.88 (2)
C4—H4	0.93	O2W—H3W	0.76 (2)
C5—N1	1.3432 (19)	O2W—H4W	0.78 (3)
C5—C6	1.469 (2)	O3—Cl1	1.4490 (12)
C6—N2	1.3448 (18)	O4—Cl1	1.4260 (12)
C6—C7	1.384 (2)	O5—Cl1	1.4236 (15)
C7—C8	1.374 (3)	O6—Cl1	1.4342 (13)
C7—H7	0.93	O7—Cl2	1.4217 (13)
C8—C9	1.376 (3)	O8—Cl2	1.4187 (12)
C8—H8	0.93	O9—Cl2	1.4503 (13)
C9—C10	1.382 (2)	O10—Cl2	1.4386 (12)
C9—H9	0.93	O9—Cu1	2.3287 (12)
			
N1—C1—C2	122.07 (16)	C10—N2—C6	119.10 (13)
N1—C1—H1	119	C10—N2—Cu1	126.53 (10)
C2—C1—H1	119	C6—N2—Cu1	114.28 (10)
C3—C2—C1	119.00 (16)	Cu1—O1W—H1W	113.8 (19)
C3—C2—H2	120.5	Cu1—O1W—H2W	117.2 (15)
C1—C2—H2	120.5	H1W—O1W—H2W	104 (2)
C4—C3—C2	119.23 (16)	Cu1—O2W—H3W	122.0 (19)
C4—C3—H3	120.4	Cu1—O2W—H4W	129.3 (17)
C2—C3—H3	120.4	H3W—O2W—H4W	104 (2)
C3—C4—C5	119.14 (16)	Cl2—O9—Cu1	130.08 (7)
C3—C4—H4	120.4	O1W—Cu1—O2W	91.60 (6)
C5—C4—H4	120.4	O1W—Cu1—N1	169.24 (5)
N1—C5—C4	121.71 (15)	O2W—Cu1—N1	93.97 (6)
N1—C5—C6	114.64 (13)	O1W—Cu1—N2	93.62 (5)
C4—C5—C6	123.65 (14)	O2W—Cu1—N2	172.19 (5)
N2—C6—C7	121.64 (14)	N1—Cu1—N2	81.83 (6)
N2—C6—C5	114.61 (12)	O1W—Cu1—O9	91.11 (5)
C7—C6—C5	123.74 (14)	O2W—Cu1—O9	81.41 (5)
C8—C7—C6	118.94 (15)	N1—Cu1—O9	98.81 (5)
C8—C7—H7	120.5	N2—Cu1—O9	92.69 (5)
C6—C7—H7	120.5	O5—Cl1—O4	111.57 (9)
C7—C8—C9	119.45 (15)	O5—Cl1—O6	109.09 (9)
C7—C8—H8	120.3	O4—Cl1—O6	110.14 (8)
C9—C8—H8	120.3	O5—Cl1—O3	108.65 (9)
C8—C9—C10	118.96 (16)	O4—Cl1—O3	108.10 (8)
C8—C9—H9	120.5	O6—Cl1—O3	109.25 (9)
C10—C9—H9	120.5	O8—Cl2—O7	110.21 (8)
N2—C10—C9	121.87 (15)	O8—Cl2—O10	108.78 (8)
N2—C10—H10	119.1	O7—Cl2—O10	110.30 (9)
C9—C10—H10	119.1	O8—Cl2—O9	109.82 (8)
C1—N1—C5	118.84 (14)	O7—Cl2—O9	109.97 (9)
C1—N1—Cu1	126.52 (11)	O10—Cl2—O9	107.71 (7)
C5—N1—Cu1	114.59 (10)		
			
N1—C1—C2—C3	−0.6 (3)	C7—C6—N2—Cu1	−178.80 (12)
C1—C2—C3—C4	−0.3 (3)	C5—C6—N2—Cu1	0.18 (17)
C2—C3—C4—C5	0.7 (3)	C1—N1—Cu1—O1W	−113.5 (3)
C3—C4—C5—N1	−0.3 (3)	C5—N1—Cu1—O1W	63.8 (3)
C3—C4—C5—C6	179.30 (16)	C1—N1—Cu1—O2W	7.58 (15)
N1—C5—C6—N2	−1.7 (2)	C5—N1—Cu1—O2W	−175.14 (11)
C4—C5—C6—N2	178.72 (15)	C1—N1—Cu1—N2	−179.04 (15)
N1—C5—C6—C7	177.30 (15)	C5—N1—Cu1—N2	−1.76 (11)
C4—C5—C6—C7	−2.3 (3)	C1—N1—Cu1—O9	89.49 (14)
N2—C6—C7—C8	0.2 (3)	C5—N1—Cu1—O9	−93.24 (11)
C5—C6—C7—C8	−178.64 (16)	C10—N2—Cu1—O1W	13.96 (14)
C6—C7—C8—C9	1.1 (3)	C6—N2—Cu1—O1W	−169.38 (11)
C7—C8—C9—C10	−0.8 (3)	C10—N2—Cu1—N1	−175.84 (14)
C8—C9—C10—N2	−0.9 (3)	C6—N2—Cu1—N1	0.82 (11)
C2—C1—N1—C5	1.0 (3)	C10—N2—Cu1—O9	−77.32 (14)
C2—C1—N1—Cu1	178.17 (13)	C6—N2—Cu1—O9	99.34 (11)
C4—C5—N1—C1	−0.5 (2)	Cl2—O9—Cu1—O1W	−13.93 (10)
C6—C5—N1—C1	179.83 (14)	Cl2—O9—Cu1—O2W	−105.38 (10)
C4—C5—N1—Cu1	−178.04 (13)	Cl2—O9—Cu1—N1	161.89 (10)
C6—C5—N1—Cu1	2.33 (18)	Cl2—O9—Cu1—N2	79.74 (10)
C9—C10—N2—C6	2.2 (2)	Cu1—O9—Cl2—O8	−52.93 (12)
C9—C10—N2—Cu1	178.71 (12)	Cu1—O9—Cl2—O7	68.52 (11)
C7—C6—N2—C10	−1.9 (2)	Cu1—O9—Cl2—O10	−171.24 (9)
C5—C6—N2—C10	177.11 (14)		

### Hydrogen-bond geometry (Å, °)

**Table:**

*D*—H···*A*	*D*—H	H···*A*	*D*···*A*	*D*—H···*A*
O1W—H1W···O10^i^	0.72 (2)	2.04 (2)	2.7078 (17)	155 (3)
O1W—H2W···O3^ii^	0.88 (2)	1.89 (2)	2.7665 (17)	177.3 (18)
O2W—H3W···O3^iii^	0.76 (2)	2.13 (2)	2.8802 (18)	169 (2)
O2W—H4W···O4	0.78 (2)	2.37 (2)	2.9518 (19)	133 (2)
O2W—H4W···O7^iv^	0.78 (2)	2.26 (3)	2.8349 (18)	132 (2)

Symmetry codes: (i) *x*−1, *y*, *z*; (ii) −*x*+1, −*y*+1, −*z*; (iii) *x*+1, *y*, *z*; (iv) −*x*+2, −*y*+1, −*z*.
